# From Molecular to Nanotechnology Strategies for Delivery of Neurotrophins: Emphasis on Brain-Derived Neurotrophic Factor (BDNF)

**DOI:** 10.3390/pharmaceutics5010127

**Published:** 2013-02-08

**Authors:** Claire Géral, Angelina Angelova, Sylviane Lesieur

**Affiliations:** 1 CNRS UMR8612 Institut Galien Paris-Sud, 5 rue J.-B. Clément, F-92296 Châtenay-Malabry, France; E-Mails: clairegeral@live.fr (C.G.); sylviane.lesieur@u-psud.fr (S.L.); 2 Univ Paris Sud 11, 5 rue J.-B. Clément, F-92296 Châtenay-Malabry, France

**Keywords:** neurotrophin BDNF delivery, therapeutic protein encapsulation, lipid nanocarriers, cubosomes, neurotrophic gene vectors, peptide mimetics, TrkB receptor, neurodegenerative disease therapy

## Abstract

Neurodegenerative diseases represent a major public health problem, but beneficial clinical treatment with neurotrophic factors has not been established yet. The therapeutic use of neurotrophins has been restrained by their instability and rapid degradation in biological medium. A variety of strategies has been proposed for the administration of these leading therapeutic candidates, which are essential for the development, survival and function of human neurons. In this review, we describe the existing approaches for delivery of brain-derived neurotrophic factor (BDNF), which is the most abundant neurotrophin in the mammalian central nervous system (CNS). Biomimetic peptides of BDNF have emerged as a promising therapy against neurodegenerative disorders. Polymer-based carriers have provided sustained neurotrophin delivery, whereas lipid-based particles have contributed also to potentiation of the BDNF action. Nanotechnology offers new possibilities for the design of vehicles for neuroprotection and neuroregeneration. Recent developments in nanoscale carriers for encapsulation and transport of BDNF are highlighted.

## 1. Introduction

### 1.1. Burden of Neurodegenerative and Psychiatric Diseases Resulting from Neurotrophin Impairment

Neurotrophic factors are proteins, which play an important role in proliferation, differentiation, maintenance, plasticity, survival and function of neurons in the central and peripheral nervous systems [1,2,3,4,5,6,7,8,9]. These neuroprotective molecules exert considerable control over the life and death pathways in cells [10,11,12,13,14,15,16,17,18,19,20,21,22,23,24,25,26,27,28,29,30,31]. They participate in local responses to various types of neuronal stressors [32,33,34,35,36,37,38,39,40,41,42,43,44,45,46,47,48,49,50,51,52,53]. In mammals, the neurotrophin brain-derived neurotrophic factor (BDNF) is a principal regulator of axonal growth and connectivity, neuronal differentiation, survival and synaptic plasticity [6,29,54,55,56,57,58,59,60,61,62,63,64,65,66,67,68,69,70,71,72,73,74,75,76,77,78,79,80,81,82,83,84,85,86,87]. It is a key molecular target in the development of drugs against neurological disorders [3,4,5,6,19,23,31,32,88,89,90,91,92,93,94,95,96,97,98,99,100,101,102,103,104,105,106,107,108]. Several studies have shown the involvement of BDNF in the pathogenesis of neurodegenerative diseases and psychiatric disorders, like depression and schizophrenia [4,6,15,16,27,37,50]. The neurotrophic actions of BDNF have been established with diverse neuronal populations [109,110,111,112,113,114,115,116,117,118,119,120,121,122,123,124,125,126,127,128,129,130,131,132,133,134,135,136,137,138,139,140,141,142,143,144,145,146,147,148]. In the periphery system, BDNF has shown neurotrophic actions on small fiber sensory neurons involved in sensory neuropathies. In the central nervous system (CNS), BDNF has been found to be a potent neurotrophic factor for cholinergic neurons, which are depleted in Alzheimer’s disease; for dopaminergic neurons of the substantia nigra, which are lost in Parkinson’s disease; as well as for cerebral and spinal motor neurons, which degenerate in amyotrophic lateral sclerosis (ALS) [12,13,14,15,16,17,18,19,20,21,22,23,24,25,26,27,28,29,30,31,32,33,34,35,36,37,38,39,40,41,42,43,44,45,46,47]. 

Despite these promising results, the therapeutic delivery of recombinant human BDNF has raised a number of problems related to its pharmacokinetic profile, short *in vivo* half-life, uncertain passage through the blood-brain barrier (BBB) and high manufacturing costs [1]. A major question has been how to assess the amount of BDNF that reaches the affected neurons, as this compound is relatively unstable and only a small fraction of it can cross the BBB after administration. If the amount of administered BDNF is too small, it may not be sufficient to produce the required neurotrophic effects. On the contrary, if the BDNF quantity is too large, it may be toxic and dangerous, because of side effects. Besides regulating the survival, maintenance and differentiation of neurons [6], BDNF also modulates the activity-dependent neuronal plasticity, which is essential for the functional and structural refinement of the neuronal circuits, as well as for learning and memory [11,25,73]. Uncontrolled BDNF administration may interfere with these mechanisms and give rise to serious side effects, such as epilepsy. It has been reported that high BDNF levels may downregulate the expression of the TrkB receptor [85,86,87], thus hampering the signaling pathways activated by BDNF and blocking any beneficial neuroprotective effects [20,49,56,63]. Therefore, it has been concluded that BDNF delivery should be localized [5] and targeted in specific brain regions, which are essential for the treatment of particular neurodegenerative diseases (Alzheimer’s disease, Parkinson’s disease, Huntington’s disease, ALS, multiple sclerosis, stroke, Rett syndrome, *etc*.). 

The rational design of neurotrophin-based therapeutics and delivery systems [53,63,149,150,151,152,153,154,155,156,157,158,159,160,161,162,163,164,165,166,167,168,169,170,171,172,173,174,175,176,177,178,179,180,181,182,183,184,185,186,187,188,189,190,191,192,193,194,195,196,197,198,199,200,201,202,203,204,205,206,207,208,209,210,211,212,213,214,215,216,217,218,219,220,221,222,223,224,225,226,227,228,229] requires a good understanding of the structures of the neurotrophic proteins and their receptors, as well as of their interactions and mechanisms of action at the molecular level. The involved biochemical structures and signal transduction pathways are briefly reviewed here, as the clinical efficiency of any neurotrophin-based drug delivery system should be evaluated by its capacity to modulate the BDNF levels and activate receptor TrkB signaling [35,36,37,38,39,40,41,42,43,44,45,46,47,48,49,50,51,52,53,54,55,56,57,58,59,60,61,62,63,64,65,66,67,68,69,70,71,72,73,74,75,76,77,78,79,80,81,82,83,84,85,86,87]. 

### 1.2. Mechanism of Neurotrophin Action

#### 1.2.1. Structure-Activity Relationships for Neuroprotection

**Figure 1:**
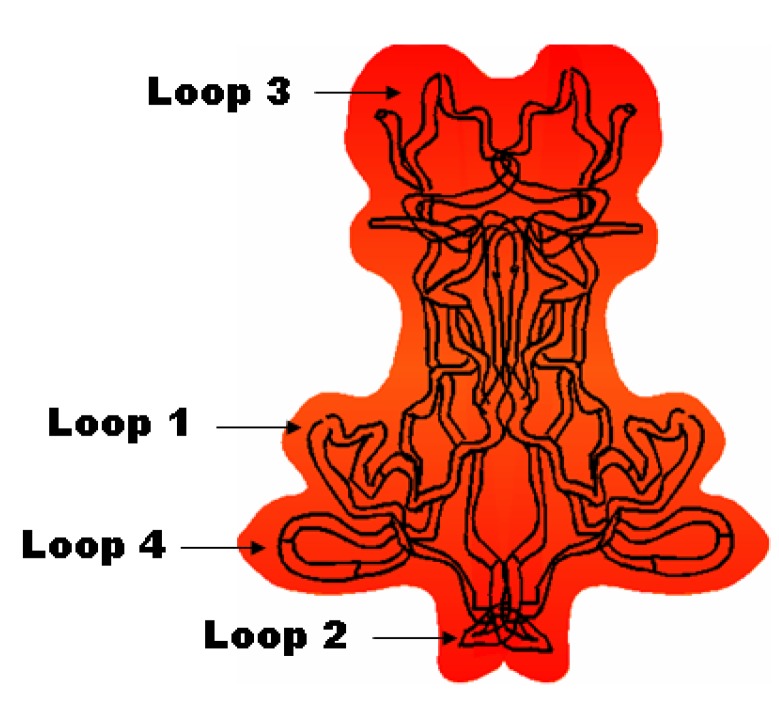
Schematic presentation of a neurotrophin (brain-derived neurotrophic factor (BDNF)) dimer with designation of the four loops participating in receptor recognition and binding.

Neurotrophic actions are exerted by four members of the neurotrophin family: brain-derived neurotrophic factor (BDNF), nerve growth factor (NGF), neurotrophin-3 (NT-3) and neurotrophin-4/5 (NT-4/5) [3,11]. Neurotrophins form homodimers of about 27 kDa and have a common structural motif, including three disulfide bridges [39,40]. Each monomeric sequence consists of approximately 120 amino acids and forms three pairs of anti-parallel β-sheets connected to four loops. The neurotrophic proteins exert their biological activity in a dimeric state (Figure 1). The amino acid residues in their β-sheets are highly conserved and are essential for the maintenance of the tertiary structure of the neurotrophins. At variance, the *N*- and *C*-terminal regions and the loops 1–4 of the proteins are variable and play a functional role in the receptor activation upon ligand binding [74,77]. 

**Figure 2:**
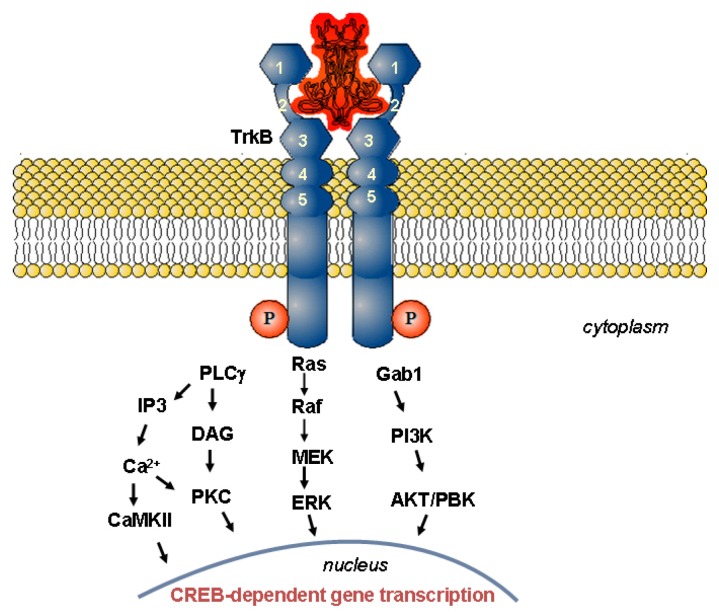
Schematic presentation of the BDNF-activated tropomyosin-related kinase B (TrkB) signal transduction. The neurotrophin binding to the TrkB receptor controls three major intracellular signaling pathways [77]. Receptor phosphorylation and activation of Ras results in activation of the mitogen-activated protein kinase (MAPK)-signaling cascade, which promotes neuronal differentiation, including neurite outgrowth and neurogenesis. Activation of phosphoinositol-3 kinase (PI3K)/protein kinase B (AKT) pathway, through Gab1, promotes survival and proliferation of neurons and other cells. Activation of phospholipase C-γ (PLC-γ) results in activation of Ca^2+^ and protein kinase C-regulated pathways that promote synaptic plasticity and neurotransmission. Each of these signaling pathways also regulates gene transcription (CREB, cAMP response-element binding protein).

The neurotrophic proteins operate by binding to two types of receptors: a common receptor, p75^NTR^, which is a member of the family of the tumor necrosis factor receptors, and a second receptor of a high affinity, the tropomyosin-related kinase (Trk) receptor, belonging to the large family of tyrosine kinase receptors [74,77]. All neurotrophic factors have the same affinity for the p75^NTR^ receptor (K_d_ ≈ 10^−9^ M), but their binding to the Trk receptors is more selective and with greater affinity (K_d_ ≈ 10^−11^ M). NGF activates the TrkA receptor; BDNF and NT-4/5 activate the TrkB receptor; and NT-3 activates the TrkC receptor. The Trk receptors have high sequence homology between them. Their extra-cellular domains have been classified into five subdomains on the basis of the sequence similarity with other known receptors: a leucine-rich region (domain 2), located between two cysteine-rich regions (domains 1 and 3) and adjacent to the two immunoglobulin-like domains (domains 4 and 5) in the proximity of the cell membrane (Figure 2). The intracellular tyrosine kinase domain is connected to the extracellular region via a toroidal membrane element. Despite the similar Trk protein structures and sequences, the receptor interactions with each neurotrophin are distinct and appear to involve a large number of discrete contacts. Variable regions, as well as slight differences between the binding sites, distinguish the neurotrophins and are important for the specificity and the binding affinity for their receptors [74]. 

The available crystallographic neurotrophin structures, BDNF/NT-3 (PDB code 1B8M) and BDNF/NT-4/5 (PDB code 1BND) [39,40], as well as the structural data for a neurotrophin co-crystallized with fragments of its receptor (PDB code 1HCF), have allowed for a better understanding of the neurotrophin-receptor interactions [77]. In particular, it has been established that the interaction of the TrkB receptor with BDNF is mediated by multiple contacts. The occurring cooperative binding activates the receptor through receptor dimerization [69]. Indeed, the amino acid structural elements, like Lysine 96 and Arginine 97 in loop 4 of BDNF and Glutamine 84 in its β sheet, as well as the residues 45–49 in loop 2 and 26–35 in loop 1, appear to be essentially important for BDNF activity, as they mediate the BDNF/TrkB contacts [69]. The TrkB receptor activation induces autophosphorylation of the intracellular tyrosine kinase domain, thereby triggering a signaling cascade to allow neurite outgrowth and neuronal survival or differentiation [77,78]. *In vitro* studies with various cell types have revealed that a complex signaling pathway is set up in the cells upon activation and dimerization of the TrkB receptor after BDNF binding [6,54,64,68,70]. Figure 2 schematically presents the signal transduction pathways, which control the neuronal cell adhesion, migration, survival, synaptic plasticity and neurogenesis upon activation or inhibition of the corresponding proteins and genes. 

#### 1.2.2. Physiological Role of BDNF in Relation to Neurodegenerative Disorders

A number of studies have presented evidence that the alteration of the BDNF levels in the CNS can cause multiple pathologies [1,2,3,4,5,6]. BDNF levels are generally decreased in the brain of patients suffering from Alzheimer’s, Parkinson’s or Huntington’s diseases [63]. Owing to the fact that this neurotrophic protein is continuously required for the maintenance and survival of mature neuronal phenotypes, it has been suggested that changes in its concentration or its distribution may lead to neurodegenerative pathologies [1]. For instance, a significant drop in the BDNF levels in the striatum has resulted in clinical manifestations of Huntington’s disease [2,17]. Studies performed on stroke, in either mice or humans, have indicated that the release of BDNF has been altered [25]. Whereas BDNF hyperactivity has been detected in epilepsy, autism and manic-depressive psychosis [49], a decreased activity of BDNF has been established in the hippocampus of patients with severe depression [6,50]. Impaired signaling, induced by BDNF, has been reported for schizophrenia [15,36]. BDNF has exerted effects also on food intake, obesity and associated metabolic conditions in animal models [13,51,55,65]. It plays a role in the mechanisms of alcohol and drug addiction [25]. 

For neurodegenerative disorders, BDNF has been validated as a therapeutic target, since the endogenous administration of the neurotrophin has produced beneficial effects [1,4,7,8,9]. BDNF promotes neuronal cell survival, adhesion, migration, neurogenesis, long-term potentiation and plasticity [25,26,27,28,29,30,31,43,81]. This neurotrophin appears to control the mechanisms of neuroprotection and memory [11,12]. Indeed, increasing of the BDNF levels has contributed to modifying the neurological disease progressions by diminishing neuronal death [1,5,63]. It has been shown that the antidepressant effects of BDNF are mediated by the MAPK/ERK and PI3K/Akt intracellular signaling pathways [59,111]. Neuroprotection has been established also through the Bcl-2/Bax-dependent mechanism, *i.e.*, by upregulation of the anti-apoptotic protein Bcl-2 and downregulation of the pro-apoptotic Bax protein upon BDNF administration [155]. 

## 2. Therapeutic Strategies Designed for Delivery of Neurotrophins

Neurotrophins are challenging candidates for drug development, because of their low bioavailability for therapeutic targets and insubstantial pharmacokinetic behavior. Generally, the use of peptides and proteins in medicine has been hampered by the short *in vivo* half-life, poor bioavailability, resulting from proteolysis or hydrolysis, and marginal permeability through biological barriers. The successful delivery of therapeutic protein molecules has required the development of carrier systems that avoid (i) rapid protein elimination from the cerebral circulation owing to enzymatic degradation, (ii) capture by the reticuloendothelial system, (iii) macromolecular accumulation in non-targeted tissues, as well as (iv) undesired immune responses. 

A starting point towards improvement of the neurotrophin drug bioavailability to brain is provided by a summary of the different therapeutic strategies for administration of such neuroprotective molecules [53,56,88,89,90,91,92,93,94,95,96,97,98,99,100,101,102,103,104,105,106,107,108,109,110,111,112,113,114,115,116,117,118,119,120,121,122,123,124,125,126,127,128,129,130,131,132,133,134,135,136,137,138,139,140,141,142,143,144,145,146,147,148,149,150,151,152,153,154,155,156,157,158,159,160,161,162,163,164,165,166,167,168,169,170,171,172,173,174,175,176,177,178,179,180,181,182,183,184,185,186,187,188,189,190,191,192,193,194,195,196,197,198,199,200,201,202,203,204,205,206,207,208,209,210,211,212,213,214,215,216,217,218,219,220,221,222,223,224,225,226,227,228,229,230]. This helps define future roads for controlled protein delivery of higher efficiency. Table 1.I summarizes the major approaches for neurotrophin delivery applied to human subjects and animal or *in vitro* models. To our knowledge, none of these approaches has resulted in a marketed formulation of pure BDNF. The commercial product, cerebrolysin^®^ (Ebewe Neuro Pharma, Unterach, Austria), appears to be a mixture involving fragments of different neurotrophic factors [157]. It has been tested in phase II clinical trials of moderate Alzheimer’s disease. 

### 2.1. Administration of Neurotrophins by Injection

Carrier-free administrations of BDNF (Table 1.I) have met little clinical success [47]. The first clinical trials have been performed with subcutaneous or intrathecal administrations of recombinant human BDNF in patients with amyotrophic lateral sclerosis (a motor neuron degenerative disease) [47]. This therapy has been well tolerated by patients, but it has lacked efficacy, due to the very short *in vivo* half-life of the therapeutic protein (<2 min) and its limited diffusion through the blood-brain barrier (BBB) [47]. For the treatment of Parkinson’s disease, minipump systems have permitted a dosage control of glial cell-derived neurotrophic factor (GDNF) in the brain, but they have provoked tissue damage and side effects (cerebellar lesions, hallucinations, development of GDNF antibodies) [119,139,159]. A direct effect of GDNF on the dopamine storage capacity and function has been demonstrated [107]. 

Preclinical studies have been performed on rat models of induced cerebral ischemia, permitting the study of the protective effects of BDNF with intraventricular pretreatment or arterial venous treatments [154,155,164]. Intravenous treatment with BDNF has significantly reduced the infarct size. The observed neuroprotection has been related to BDNF-dependent downregulation of the pro-apoptotic protein, Bax, and upregulated expression of the anti-apoptotic protein, Bcl-2, in neurons in the ischemic penumbra. To study the influence of BDNF on long-term memory storage, a study has been performed using intrahippocampal neurotrophin injection in rats [11]. BDNF delivery has compensated the inhibited hippocampal protein synthesis and has induced memory persistence through ERK-signaling. On the other hand, BDNF administration via cochlear implants of osmotic mini-pumps has shown prolonged effects throughout deafness treatment in guinea pigs, even after two weeks of the discontinued infusion [88]. Owing to the fact that the *in vivo* half-life of the protein in the CNS is short, as a result of high turnover of the cerebrospinal fluid, the possibilities for neurotrophin diffusion to target cells have been limited [47,139]. Therefore, other approaches have searched for effective delivery of optimal BDNF doses.

### 2.2. Neurotrophin Gene and Cell Therapy

Local delivery of neurotrophins to neurons has been performed by gene therapy [12,32,41,118,140,161,184,185,186,187,188,189,190,191,192,193,194,195,196,197,198,199,200,201,202,203,204,205,206,217]. This method avoids the protein instability in circulation and may yield a lasting expression of BDNF [2]. Neurotrophin gene therapy (Table 1.II) has given encouraging pre-clinical results in studies of Huntington’s disease and multiple sclerosis with rodents [12,194,197,200]. The intrastriatal injection of BDNF-encoding adenovirus has been established to be neuroprotective in an animal model [12,194]. Regulation of the locally produced neurotrophin levels has been achieved by intranigral implants secreting the protein [161]. In addition, a clinical trial for gene therapy of Alzheimer’s disease has been performed with *ex vivo* transfected fibroblasts using viral vectors encoding for the human NGF gene [203]. The gene delivery system has been a graft of autologous fibroblasts genetically modified to express human NGF into the forebrain. Lentivirus and adenovirus types of vectors have been used for the transfer of NGF and NT-3 encoding genes in rat models of spinal cord injury [41,118,165,199,217]. Viral vectors, inducing long-lasting expression of neurotrophin molecules, have been studied in a rat model of epilepsy [140], whereas lentivirus systems, secreting BDNF, have been applied to models of Alzheimer’s disease in rodents and primates [32]. Increased neurogenesis and broad neuroprotective effects have been observed [32,41,165]. The toxicity and inflammation caused by certain viral vectors, the risk of accidental tumor formation by mutagenesis and the invasive approach of this method have created challenges for its long-term clinical application with patients [1]. 

To avoid viral delivery of BDNF into the brain, alternative therapeutic strategies have been proposed for transplantation of cells releasing the neurotrophin [188,196,225]. Bone marrow stem cell (BMSCs)-based BDNF gene delivery into the CNS has shown a therapeutic effect on the reduction of the severity of disease by decreasing inflammation and enhancing immunomodulation and remyelination [197]. TrkB receptors have been activated by BDNF released by human mesenchymal stem cells (hMSC) implanted intranigrally to regulate neurotrophin expression [161] with a potential application in Parkinson’s disease therapy. It should be stressed that these methods present a risk of rejection and can cause accidental tumor growth. Another approach for treatment of CNS lesions has employed non-viral vectors for transfer of genes encoding for BDNF and NGF [195]. 

**Table 1:** Summary of therapeutic strategies for treatment with neurotrophins *^a^*.

Therapeutic strategy	Neurotrophin	Application	Model	Outcome	Refs.
**I. Administration of recombinant proteins by direct injection**					
Subcutaneous and intrathecal injection	BDNF	Amyotrophic lateral sclerosis (ALS)	clinical trial phase III	limited BDNF diffusion through the BBB; high dose required to observe survival effects	[47]
Intracerebroventricular infusion via implanted catheters	GDNF	Parkinson’s disease	clinical trials phase I	GDNF did not reach substantia nigra; side effects	[119,139]
Direct intraputamenal perfusion via implanted mini-pumps	GDNF	Parkinson’s disease	clinical trials phase I and phase II	clinical improvement of symptoms after 1 year of therapy; GDNF effect on dopamine function	[107,124,159]
Intraventricular pretreatment	BDNF	Cerebral ischemia	rat	reduced infarct size	[154]
Intraventricular infusion pumps	BDNF	Cerebral venous ischemia	rat	reduced infarct size; protection of cerebral cortex against apoptosis	[164]
Intravenous	BDNF	Cerebral ischemia	rat	reduced infarct volume	[155]
Mini-pump in the cerebral artery	BDNF	Cerebral ischemia	rat	reduced infarct size	[178]
Intra-hippocampal injection	BDNF	Long-term memory (LTM) storage	rat	memory persistence	[11]
Cochlear implant of osmotic mini-pump	BDNF	Deafness	guinea pig	enhanced survival of auditory nerves	[88]
Intracerebroventricular infusion (ICV)	NGF	Alzheimer’s disease	rodents, clinical trials	increased number of axons; prevented degeneration of cholinergic neurons	[220,221,222,223]
Intranasal	BDNF; NT-4	CNS disorders; Cerebral ischemia	rat	noninvasive delivery; minimal systemic exposure; enhanced neurogenesis; unknown pharmacokinetics	[53,91,129]
ICV administration; protein infusion	BDNF	Dependence on psychostimulants	rat	long-lasting antidepressant effects by the use of molecules activating the PI3K/Akt and MAPK/ERK pathways; neuroplasticity	[59,111]
**II. Gene and cell therapy**					
Gene transfer via adeno-associated viral (AAV) vector	BDNF; GDNF	Huntington’s disease	rat; mice	striatal neuron survival	[12,194,200]
*Ex vivo* gene delivery by genetically modified fibroblasts secreting the protein	NGF	Alzheimer’s disease	clinical trial Phase I	cholinergic neuron stimulation; modified disease progression	[203,204]
Gene transfer via lentivirus or adenovirus followed by protein expression	BDNF	Alzheimer’s disease	mice; rats; monkeys; clinical trials	broad neuroprotective effects	[5,32]
Lentiviral vectors for local delivery in gene therapy	BDNF; NT-3	Spinal cord injury	*In vitro*; rats	bridging axonal regeneration across lesion sides	[165,217]
Gene transfer via adenovirus	NGF; BDNF	Spinal cord injury	rats	axonal regeneration and collateral sprouting; axonal growth	[41,118,199]
Herpes simplex virus induced long lasting protein expression	BDNF	Epilepsy	rats	increased neurogenesis; reduced epileptogenesis	[140]
Gene transfer via cationic liposomes	BDNF; NGF; GDNF	CNS lesion; Spinal cord injury	*In vitro*	transgene expression at low cellular toxicity	[179,195]
Gene transfer via genetically-engineered bone marrow stem cells expressing the protein	BDNF; GDNF; NGF; CNTF	Multiple sclerosis; Huntington’s disease; spinal cord injury; glaucoma	mice; rats; *In vitro*	suppressed demyelination; reduced motor dysfunction; decreased inflammation	[114,196,197,201]
Transplants of genetically-engineered fibroblasts expressing the protein	BDNF; NGF; NT-3	Parkinson’s disease; Spinal cord injury	rats	increased nigral dopaminergic neuronal survival responsiveness to axonal regeneration	[103,127,137]
Neural stem cell transplantation	BDNF	Alzheimer’s disease	mice	improved cognitive function	[224]
Encapsulated cell biodelivery (ECB) -implanted device with encapsulated protein-secreting cells	NGF	Alzheimer’s disease	Göttingen mini-pigs; clinical trials	persistent NGF secretion; increased neurotrophin levels in the basal forebrain; safety and tolerability; new therapeutic platform in restorative neurosurgery	[102,225]
*Ex vivo* gene therapy via protein-expressing BHK cells encapsulated in a device with a semi-permeable polymer membrane	CNTF	Huntington’s disease	clinical trial Phase I	proof of principle for implanted capsules	[184,188]
Intranigral transplants of mesenchymal stem cells secreting the protein	BDNF	Parkinson’s disease	*in vitro*; rats	regulated BDNF expression and dopaminergic effect	[161]
**III. Sustained release using polymer systems *III.A. Synthetic polymers***					
Polyethylene glycol (PEG) chain conjugated at the *C*-terminus of the recombinant protein (intravenous administration)	BDNF	unspecified	*in vitro*; rats	retained bioactivity of PEGylated neurotrophin	[149]
Conjugation of a PEG chain and an OX26 antibody (biotin/SA) for targeted delivery (intravenous administration)	BDNF	Cerebral ischemia	*in vitro*; rats	increased brain uptake of the BDNF construct; minimized rapid clearance upon PEGylation	[141,175,183]
Covalent coupling with PEG chains (intrathecal injection)	BDNF	Spinal cord injury and diseases	*in vitro*; rats	improved half-life in the cerebrospinal fluid; increased effect on locomotor activity	[89,160]
PLGA-PLL-PEG biodegradable microspheres releasing recombinant protein	BDNF	CNS injury	*in vitro*	sustained release of bioactive human BDNF over 60 days	[94]
PLGA particles dispersed in a hydrogel of hyaluronic acid (HA) and methylcellulose	Chymotrypsin as a model of NT-3 and five other neuroprotectors	Spinal cord injury	*in vitro*	sustained release over 28 days from injectable composite hydrogels	[95]
Poly(lactic-co-glycolic acid) (PLGA) microspheres releasing recombinant protein	NGF	Huntington’s disease; unspecified lesions	rats; *in vitro*	sustained release over 2.5 months; improved protein stability; reduced striatal lesions	[123,134,143]
PLGA biodegradable microspheres releasing recombinant protein	GDNF	Parkinson’s disease	*in vitro*; rats; monkeys	improved dopaminergic graft survival and function	[90,98,104,105,106,107,115,116]
Ethylene-co-vinyl acetate (EVAc) discs for sustained release	NGF	Alzheimer’s disease	*in vitro*; rats	controlled release for up to one week; limited NGF transport in the brain tissue; high concentrations near the implant	[121]
EVAc discs and PLGA microspheres	NGF	unspecified CNS disease	*in vitro*; rats	high localized doses of recombinant protein near the implants; half-life increased to 1.7 hours	[153]
PLA-PEG hydrogel	BDNF; NT-3; NGF	Spinal cord or optic nerve injury	*in vitro*; mice; rats	sustained release over 2 weeks; simultaneous delivery of multiple neurotrophins; stimulated proliferation; enhanced neurite outgrowth	[97,145,173]
Polyphosphoester (PPE) microspheres incorporated in nerve guide conduits	NGF	Nerve injury	rats	morphological regeneration of sciatic nerve 3 months after the implantation of the conduits	[177]
Ethylene-vinyl acetate (EVA) nerve guidance channels releasing the protein	GDNF; NGF; NT-3; BDNF	Sciatic nerve injury	*in vitro*; rats	promoted regeneration of myelinated axons	[92,96,101]
Poly(lactide-co-glycolide) (PLG) microspheres in nerve guide conduits	NGF	Spinal cord and peripheral nerve injury	*in vitro*; mice	sustained release over 42 days from the porous constructs allowing for cellular infiltration into the channels; stimulated neurite outgrowth	[180]
PLA tubular macroporous foam	BDNF	Spinal cord injury	*in vitro*; rats	low axonal regeneration response; increased angiogenesis	[142]
Macroporous scaffold of pHEMA and PLL	NGF; NT-3	Nerve injury	*in vitro*	minimum concentration gradient of 200 ng/mL required for guidance of the neurite outgrowth	[136]
Conducting polypyrrole scaffold with surface-conjugated proteins	NGF; NT-3	Nerve injury	*in vitro*; rats	nerve fiber growth towards the implant electrode	[108,146,169]
Implanted EVAc matrices	BDNF	Major depression	rats	dysregulation of BDNF-associated plasticity-related pathways upon sustained release; antidepressant-like effects upon short-term delivery	[158]		
***III.B. Naturally occurring polymers***					
Alginate microspheres	NGF; BDNF	Brain injury; major depression	rats	prevented neuronal degeneration; release over 1-2 days; antidepressant-like behavioral effects of BDNF	[132,158]
Agarose hydrogels	BDNF	Spinal cord injury	rats	encouraged neurite growth into the channels; axonal regeneration; minimal inflammatory response	[113,163]
Protein bound to collagen in linearly ordered collagen scaffolds (LOCS)	BDNF	Spinal cord injury	*in vitro*; rats	improved neuron survival and recovery of spinal cord injury	[110]
Hyaluronic-acid hydrogel scaffold	BDNF	Spinal cord injury	*in vitro*; rats	regeneration; improvement in locomotive tests	[218]
Agarose hydrogel coupled with laminin	NGF	Nerve injury	*in vitro*	enhanced neurite extension	[182]
Collagen matrix implants	NT-3	Spinal cord injury	rats	attraction of corticospinal tract fibers into the graft; recovery function	[112]
Fibrin matrix containing heparin (or peptide) bound via electrostatic interactions to recombinant protein	BDNF; NGF;NT-3	Unspecified; Spinal cord injury; Sciatic nerve injury	*in vitro*; rats	enhanced nerve regeneration across short nerve gaps; localized controlled release up to 7 days; dose-dependent axonal regeneration; affinity-based delivery systems for neural tissue engineering	[126,131,150,166,167,171]	
**IV. Lipids and diets variations**					
Caloric restriction; physical exercise	BDNF; GDNF	Parkinson’s disease	Rhesus monkeys	higher locomotor activity; increased neuronal survival in substantia nigra and striatum	[125,130]
Potentiation by omega-3 fatty acid	BDNF	cellular model of neurodegeneration	*in vitro*	increased cell survival	[219]
Triglyceride matrix implants	BDNF (lysozyme model)	Huntington’s disease	*in vitro*; rats	controlled release over 1-2 months; preserved protein activity and integrity	[120]
**V. Peptidomimetics, small molecule mimetics and prodrugs**					
Peptidomimetics	BDNF	Neurodegenerative disorders	*in vitro*	BDNF-like agonist action; sensory neurons survival	[207,208,209,210,211]
Small molecule mimetics and modulators	BDNF	Motor trauma; Alzheimer’s disease	rodents; *in vitro*	TrkB agonists; modulation of the activity of the TrkB receptor; improved motor learning; promoted neurogenesis	[212,213,214,215,216]
Prodrugs of non-peptide neurotrophin mimetics	non-peptide mimetics of BDNF	Psychiatric disorders	Mice	reduced depression- and anxiety-related behaviors; blood-brain barrier penetration	[213,215]
Peptidomimetics	NT-3	Peripheral neuropathies; neurodegenerative diseases	*in vitro*; animal models	selective inhibition of TrkC-mediated cell survival; neuroprotection	[226,227,228]

^a^ BDNF: brain-derived neurotrophic factor; GDNF: glial cell line-derived neurotrophic factor; NGF: nerve growth factor; CNTF: ciliary neurotrophic factor; NT-3: neurotrophin-3.

### 2.3. Sustained Release of Neurotrophic Factors by Means of Polymer Carriers

Synthetic and natural polymers are suitable for controlled delivery of neuroprotective molecules to therapeutic targets (Table 1.III). Several studies have attempted to realize sustained release of neurotrophins to brain using a variety of polymers [90,91,92,93,94,95,96,97,98,99,100,101,102,103,104,105,106,107,108,109,110,111,112,113,114,115,116,117,118,119,120,121,122,123,124,125,126,127,128,129,130,131,132,133,134,135,136,137,138,139,140,141,142,143,144,180,181,182,183]. 

#### 2.3.1. Synthetic Polymers

One of the first polymeric formulations, designed for intracerebral implants releasing NGF, has employed the nondegradable polymer poly(ethylene co-vinyl acetate) (EVAc) [121]. Some years later, delivery systems have been fabricated from EVAc discs and poly(lactic-co-glycolic acid) (PLGA) biodegradable microspheres and have been compared in *in vitro* and *in vivo* studies in rats [153]. The controlled delivery of NGF from intracerebral implants, built-up from PLGA microspheres, has been extensively studied *in vitro* and *in vivo* [123,134,143]. Higher local concentrations of recombinant protein have been observed near the implants. Controlled local release of GDNF from biodegradable PLGA microspheres has been realized with rats subjected to brain stereotaxy for local intracranial microspheres implantation [90,98,104,105,106,115,116]. The comparison of different delivery systems has indicated distinct patterns of recombinant protein (rhNGF) release in rats (for 10 mg intracranial implants), *i.e.*, (i) sustained release from ethylene-co-vinyl acetate (EVAc) discs, (ii) immediate release from poly(lactic-co-glycolic acid) (PLGA) microspheres prepared by double emulsion/solvent evaporation and (iii) delayed release from PLGA microspheres prepared by spray freeze-drying. The latter system has provided the highest release rate. The diffusion coefficient of rhNGF through the brain tissue (8 × 10^−7^ cm^2^/s) has been evaluated to be ~50% of the diffusion coefficient in water solution. The achieved rates of rhNGF release, greater than 100 ng per day, have been appropriate for treating animal models of Alzheimer’s disease [153]. 

Biodegradable microspheres of the triblock polymer PLGA-polylysine-polyethylene glycol (PLGA-PLL-PEG) have provided sustained release of BDNF during 60 days of *in vitro* assays [94]. The encapsulated BDNF has preserved its bioactivity. The PLGA-PLL-PEG microspheres have yielded greater loading and longer-term delivery of BDNF, as compared to the PLGA ones. It has been suggested that this amphiphilic polymer increases the interaction of the neurotrophin with the carrier and leads to sustained release that closely correlates with the polymer degradation [94]. On the other side, special hyaluronan (HA) and methylcellulose (MC) hydrogels, containing PLGA particles releasing six different neuroprotective proteins, have been administered by intrathecal injections [95]. Biodegradable hydrogels formed by the copolymer of polylactic acid (PLA) and polyethylene glycol (PLA-PEG) have also been studied as neurotrophin carriers [97,145,173]. The outcomes of these studies with animal models are summarized in Table 1.III.

Microspheres of polyphosphoester (PPE), containing NGF, have been transferred into synthetic nerve guidance conduits allowing for sustained neurotrophin delivery and regeneration of damaged neurons [177]. Nerve guide channels have been synthesized from EVAc as well [92,96,101]. Copolymers of lactic and glycolic acids (PLG) have served for the construction of nerve conduits releasing neurotrophin [180]. Macroporous foams of PLA have been used as brain implants for local release of BDNF [142]. Scaffolds of polyhydroxyethyl (pHEMA) and polylysine (PLL) have ensured defined concentration gradients of NGF and NT-3 [136]. Work has been done also on coupling of neurotrophins on the surface of the biocompatible polymer polypyrrole, which has electric conductive properties [108,146,169]. The advantage of the polymer materials implanted into the CNS (PLG, PLA, PLL, EVAc, pHEMA, PPE) is their biocompatibility. The nature of the polymers influences the adsorption of proteins and the extent of adhesion of inflammatory cells on the implants. The shape and the surface area of the implants (microspheres with high surface area *versus* large-sized conduits with small material/tissue interfaces) also determine the inflammatory response. Brain tissue reactions to the scaffolds implantation have often involved acute inflammatory response within the first week in relation to the mechanical damage to the CNS, rather than to the nature of the polymer material. 

The PEGylation of the *C*-terminus of BDNF, via conjugation of a polyethylene glycol (PEG) chain, has been efficient in reduction of the systemic clearance of the neurotrophin and has resulted in minimal loss of its biological activity [149]. BDNF, covalently conjugated to one or more PEG chains, has also preserved its biological activity with enhanced distribution in the spinal cord [160]. In affinity-based systems, the biotinylation of the PEG terminus of the PEG-modified neurotrophin has served for its coupling to the anti-transferrin monoclonal antibody OX26 (MAb OX26) through an anchored streptavidin (SA). The created protein construct has allowed for activation of the transferrin receptor, which is abundantly present in the brain capillary endothelium. The BDNF-PEG-MAb-OX26/SA conjugate has been employed for BDNF targeting to brain after intravenous administration [141,175,183]. The transferrin receptor has mediated its transport through the BBB. It should be noted that the capacity of this system for BDNF delivery has been generally low, because every therapeutic protein molecule (BDNF) requires as a vector, in this receptor-mediated delivery approach, a bound monoclonal antibody molecule (OX26) (*i.e.*, one carrier entity can transport only one therapeutic molecule). At variance, Trojan horse liposomes [231,232], coupled with Mab OX26, can encapsulate several neurotrophin molecules in their aqueous reservoirs. This augments the delivery capacity of the system, as every carrier can transport many BDNF molecules. 

#### 2.3.2. Natural Polymers

Owing to its biocompatibility, biodegradability, low toxicity and hydrophobicity, alginate has been used in the form of microspheres to provide sustained release of the neurotrophin NGF [132]. Porous hydrogels of agarose have been employed for construction of scaffolds for controlled release of BDNF and NGF [113,163]. Biocompatible agarose gel scaffolds, coupled with different laminin oligopeptides, have promoted an enhanced neurite growth [181,182]. Collagen has also been a good matrix for sustained local release of neurotrophins [112]. Matrices involving fibrin derivatives and heparin-binding peptides, coupled to neurotrophins, have been extensively studied *in vitro* and *in vivo* [126,131,151,152,153,166,168,171]. The *in vitro* neurite growth, observed upon controlled protein release, has been considerable with these systems. Sciatic nerve regeneration has been achieved *in vivo* in a rat model of spinal cord injury [166,168,171]. The results obtained with fibrin matrices have indicated the role of the neurotrophin carrier serving as a therapeutic material to enhance peripheral nerve regeneration through nerve guide tubes. It has been demonstrated that BDNF, NGF and NT-3, interacting with fibrin matrices containing a large molar excess of heparin relative to the neurotrophic growth factor, may enhance neurite extension by up to 100% relative to unmodified fibrin [150]. At variance, free neurotrophins have not enhanced neurite extension in the absence of a delivery system. For the release characteristics of the natural polymer biomaterials, as biodegradable compounds of different kinetics of degradation in the tissues, the reader is referred to the cited references. 

### 2.4. Dietary Restrictions

Preclinical studies have shown that dietary restrictions and physical exercise may increase the BDNF levels [1]. In this way, multiple pathways and molecular mechanisms can be activated, permitting correction of the deficiency of neurotrophic factors. It has been reported that a low-calorie diet can attenuate the severity of neurochemical deficits and motor dysfunction in a primate model of Parkinson’s disease [130]. Understanding of the relationship between diet, energy intake and BDNF levels may thus lead to new therapeutic approaches [125,130]. For example, physical exercise has induced BNDF expression in several brain regions and has reduced the neuronal damage [1,130].

### 2.5. Peptide Mimetics of BDNF

Increased endogenous BDNF levels and neuroprotection can be achieved by small medicinal drugs [212,213,215,216]. The observed effects have generally been nonspecific. As an alternative, an emerging therapy with synthetic peptide mimetics of BDNF may offer high selectivity and specificity for the disease mechanisms. Notably, peptidomimetics can be resistant to degradative enzymes. This aspect helps in increasing the stability of the peptidomimetic drugs and their bioavailability [229]. 

**Figure 3:**
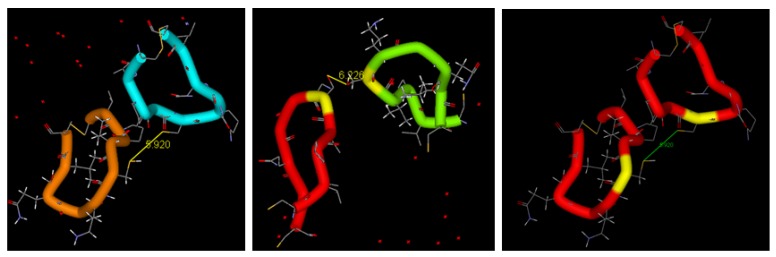
Design of peptide mimetic dimers of neurotrophic factors based on the structure of the loop 2 in heterodimer configurations BDNF/NGF (**left** and **middle**) and in a BDNF homodimer (**right**). The cysteine bridges and the interloop distances are indicated.

The peptidomimetics approach considers the three-dimensional (3D) structure of BDNF used for identification and modeling of the loops participating in binding to the TrkB receptor and its activation [207,208,209,210,211]. Towards that aim, the biological functions of the BDNF loops (Figure 1) have been examined. Site-directed mutagenesis analysis has revealed that the ability to bind and activate the TrkB receptor may be conferred by replacement of selected amino acid residues in loop 2 of NGF with those corresponding to BDNF [69]. Having in mind the established receptor-binding roles of loops 1, 2 and 4 of BDNF, mono- and bi-cyclic peptide mimetics of BDNF have been synthesized, including homodimers of the loops 2 and 4 [207]. The homodimeric bicyclic mimic of loop 4 of BDNF has been less efficient, as compared to the homodimer mimic of loop 2 (Figure 3). 

By comparison of the biological activities of designed cyclic peptide dimers with various compositions, mimicking the loops 1, 2 or 4 of BDNF, it has been concluded that the BDNF-like partial agonist activity should be related to the ability of the new compounds to dimerize the TrkB receptor in a manner similar to the native neurotrophic factor (Figure 2). Preliminary studies have shown that the designed BDNF peptidomimetics can act as BDNF agonists, promote neurite outgrowth and support the survival of sensory neurons [207,211]. In fact, peptides with conformational constraints, mimicking loop 2 of BDNF, have inhibited the neurotrophin-mediated survival of sensory neurons in culture, presumably by exerting a competitive TrkB antagonist action [210].

## 3. Soft Nanotechnologies and Nanocarrier-Mediated Delivery

Soft nanotechnology refers to design, engineering and manipulation of soft materials towards the creation of structures (such as nanoparticle objects as drug carriers) and devices of a nanometer-length scale. The majority of known nanoparticulate systems for drug delivery and targeting to the CNS [230,233,234,235,236,237,238,239,240,241] have not been exploited for neurotrophin delivery yet. A variety of nanocarriers, such as dendrimers, nanospheres, solid lipid nanoparticles (SLN), nanoemulsions, polymeric micelles, multifunctional nanoparticles (NPs) and nanoscale systems for imaging [242,243,244,245,246], are expected to be studied for controlled release of neurotrophins. Nanoparticulate systems for growth factor delivery have attracted increasing recent interest [122,176,219,231,232,246,247,248,249,250,251,252,253,254,255,256,257,258,259]. 

In searching for innovative therapeutic approaches to treat neurodegenerative disorders, colloidal nanoparticle carriers have been designed as reservoirs for neurotrophins, ensuring their protection against enzymatic degradation and other destructive stressor factors. Indeed, the walls of the nanoparticulate containers (such as lipid membrane-type vehicles, nanocapsules and nanospheres) can completely isolate the therapeutic substances from the environment. Colloidal nanocarriers can be coated with hydrophilic polymers, such as polyethylene glycol (PEG), or with albumin in order to reduce their opsonization and increase their *in vivo* circulation time. Importantly, neurotrophin encapsulation in NPs may help locally administer a neuroprotective agent in a concentrated state to target sites, while minimizing eventual systemic side effects and toxicity. Another advantage of the NPs delivery systems is the possibility to combine different types of active molecules in one nanocarrier (for instance, neurotrophic drug molecules together with a diagnostic imaging agent for cerebral theranostics). Moreover, targeted delivery can be performed via modifying the surface of the nanocarriers by anchoring of specific ligands for receptor recognition [176,248]. 

Table 2 presents examples of neurotrophin delivery systems issued by soft nanotechnologies (nanocarriers prepared by “bottom-up” self-assembly or by “scaling-down” fragmentation of bulk soft materials), as well as by nanobiotechnology (nanocarriers based on fusion proteins produced via biotechnology techniques) [122,176,219,231,232,247,248,249,250,251,252,253,254,255,256,257,258,259]. 

**Table 2:** Nanoscale carrier systems for neurotrophin delivery.

Nanosystem	Neurotrophin	Disease/Model	Reference
Polysorbate-coated poly(butyl cyanoacrylate) (PBCA) NPs	NGF	Parkinson’s disease/mouse	[122]
Nanoporous poly-L-glutamic acid (PGA) particles	BDNF	Deafness/guinea pig, *in vitro*	[254]
Layer-by-layer (LbL) films on agarose hydrogel scaffolds	BDNF (a lysozyme model)	Spinal cord injury/*in vitro*	[247]
Poly(ethylene glycol)-poly(ε-caprolactone) (PEG-PCL) polymersomes conjugated with OX26 MAb	NC-1900 peptide (an arginine-vasopressin fragment analogue)	Learning and memory impairments/rat	[248]
PEG-b-PCL polymersomes with surface-attached polyethylene glycol (PEG) chains	NGF mimetic peptide (hNgfEE) as an alternative of BDNF	*In vitro*	[255,256]
Targeted liposomes	NGF	Alzheimer’s disease/*in vitro*	[176]
Immunoliposomes	Model plasmids (luciferase, β-galactosidase, SV40-lacZ)	Brain disorders/rhesus monkey	[231,249,250,260]
Cationic liposomes	Plasmid encoding for *GDNF* or *NGF*	Spinal cord injury/*in vitro*	[179,195]
NTS (neurotensin)-polyplex nanocarrier	Neurotrophic genes (*GDNF*, *NRTN*, *BDNF*)	Parkinson’s disease/transfected dopaminergic neurons *in vitro*, rat	[205]
PEGylated cationic lipid NPs	Plasmid encoding for *BDNF*	*In vitro*	[257,258]
Cubosome NPs containing essential omega-3 fatty acid	BDNF	*In vitro*	[219]
Cubosome NPs	Neuroprotective peptide Gly14-humanin	Alzheimer’s disease/rat	[259]
Trojan horse nanocarriers	*GDNF*; plasmid driven by brain-specific promoter	Parkinson’s disease/rodents, rhesus monkeys	[231,232,245,260]
Fusion protein vectors	BDNF-IgG (OX26); NGF-IgG; GDNF-Tat	Ischemial stroke, Parkinson’s disease, Alzheimer’s disease/rats	[231,249,250,251,252,253,261]

Nanoparticles of poly(butyl cyanoacrylate), carrying NGF neurotrophin, have been characterized by enhanced penetration through the BBB after surface functionalization by polysorbate 80 [122]. BDNF has been encapsulated in nanoporous poly(L-glutamic acid) (PGA) particles, produced via mesoporous silica templating, from which it has been released in a sustained manner with retained biological activity on SH-SY5Y cells [254]. *In vivo* experiments have demonstrated that the released BDNF can efficiently rescue auditory neurons in the cochlea of guinea pigs with sensorineural hearing loss [254]. Another nanostructured system for sustained release of BDNF (using lysozyme as a model protein) has been fabricated by alternating assembly of poly(ethylene glycol)(PEG)/poly(acrylic acid)(PAA)/protein layer-by-layer (LbL) thin films on agarose hydrogel template [247]. The achieved month-long sustained protein release from the agarose hydrogel scaffold has shown promise to promote axonal regeneration in the CNS after spinal cord injury. 

A short peptide mimetic of NGF (with a sequence similar to BDNF) has been conjugated to the surface of polymersome nanoparticles and has been *in vitro* studied for TrkB receptor targeting on SHSY-G7 cells [255]. The PEGylated surface of the polymersomes has allowed for increased *in vivo* retention time of the carriers, which have acted as scaffolds for the delivery of TrkB-activating ligands. The TrkB receptor has been found to be phosphorylated when targeted by NGF-peptide-conjugated polymersomes, whereas no TrkB phosphorylation has been detected in SHSY-G7 cells incubated with unfunctionalized polymersomes [255]. This strategy may be classified as peptide-targeted nanocarriers to treat neurodegeneration. The synthetic neurotensin (NTS)-polyplex nanocarrier system has enabled delivery of neurotrophic genes to dopaminergic neurons via NTS receptor-mediated endocytosis mechanism of internalization [205]. 

**Figure 4:**
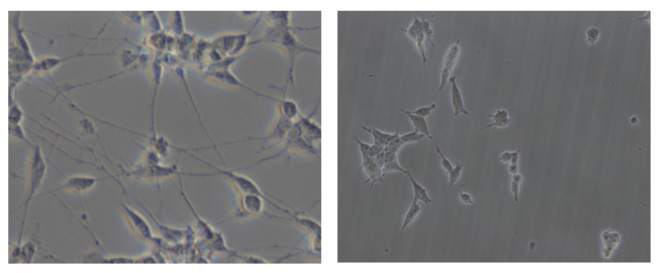
Phase contrast microscopy images of neurite outgrowth in differentiated, alive human neuroblastoma SH-SY5Y cells after neuroprotective treatment with 2 ng/mL BDNF and lipid nanoparticles (MO/DOPE-PEG_2000_/EPA, 83/2/15 mol%) with a total lipid concentration of 4 × 10^−^^7^ M (**left**), as compared to untreated SH-SY5Y cells grown in culture medium (**right**). Image size: 270 × 200 µm^2^.

In a recent work, Géral *et al.* [219] have shown the potentiation effect of multicompartment lipid carriers containing omega-3 polyunsaturated fatty acid, the eicosapentaenoic acid (EPA), on the BDNF activity in *in vitro* neuroprotective experiments with a SH-SY5Y cell line. The human neuroblastoma SH-SY5Y cells have been differentiated by retinoic acid, which has induced the expression of the TrkB receptor. The viability of the SH-SY5Y cells after five days of treatment with retinoic acid (10 μM) has been estimated to be 44% ± 6%, as compared to untreated cells (100% viability). This result has indicated an essential decrease in the cellular proliferation in the absence of neuroprotective treatment. By comparison of the relative cellular viability, determined in the presence of BDNF and multicompartment lipid NPs of a cubosome type, it has been established that the lipid composition of the nanovehicles can be very essential for neurogenesis [219]. The results have confirmed that the activity of BDNF is potentiated by the omega-3 fatty acid (EPA) rather than by the other lipids constituting the NPs. The percent of cell survival has increased almost three-times in the presence of EPA-containing NPs (at a total lipid concentration of 4.2 × 10^−8^ M). Very long and branched neurites have been observed when the neuroblastoma cells have been treated with BDNF in the presence of lipid nanocarrier systems containing EPA (Figure 4). The polyunsaturated fatty acid, EPA, has shown a real effect of potentiation of the neurotrophic factor BDNF alone and in a combination with liquid crystalline monoolein (MO)-based multicompartment nanoassembles [219]. 

Immunoliposomes, *i.e.*, PEGylated liposomes coupled with antibodies, such as the anti-transferrin monoclonal antibody (OX26 MAb), have been employed in receptor-mediated gene delivery to the brain through the transferrin receptor at the BBB [231,249,250]. PEGylated immunoliposomes have been *in vivo* targeted to the rhesus monkey brain with a monoclonal antibody to the human insulin receptor (HIR MAb) as well. The MAbs have enabled the liposomes to undergo transcytosis across the BBB and endocytosis across the neuronal plasma membrane following intravenous injection. This approach is related to the Trojan horse technology for neurotrophin targeting and release [231,232,245]. 

Receptor-mediated delivery of neurotrophins through the transferrin and insulin receptors has also been done by fusion proteins engineered for brain targeting [261,260]. The molecular Trojan horse technology involves genetically engineered chimeric complexes of neurotrophins (BDNF, NGF, GDNF) and human immunoglobulin (IgG) monoclonal antibodies recognizing the human transferrin or insulin receptors, which enable delivery across the BBB. The IgG fusion proteins have shown low immunogenicity in primates [252]. The BDNF-IgG fusion protein has demonstrated a significant neuroprotective effect against forebrain ischemic injury in rats after intravenous administration. This nanobiotechnology approach, which is alternative to the chemical conjugation strategies [141,183], has revealed a potential for clinical use in stimulation of brain repair in post-ischemic therapy [253]. 

Among the nanocarrier systems with controlled-release and biocompatibility properties, lipid nanoparticles [262] represent a very interesting way for neurotrophin administration, as well as for development of neuroregenerative therapeutics. To date, lipids of various types have been widely used for delivery of nanomedicines by different routes of administration. In the presence of an aqueous medium, amphiphilic lipids self-organize in compartmentalized structures with separated polar and apolar domains [262,263,264,265,266,267,268,269,270,271,272,273,274,275,276,277,278,279,280,281,282,283,284,285,286,287,288,289,290,291,292]. Hydrophobic interactions play a key role in stabilizing the nanostructures that form based on the existing lipid polymorphism [281]. Lipids can adopt different structural organizations, the most common ones being the lamellar bilayer, inverted cubic, inverted hexagonal, sponge and micellar phases. The diversity of the lipid/water structural arrangements is essential for the encapsulation capacity of the created carriers for therapeutic molecules [271]. 

**Figure 5:**
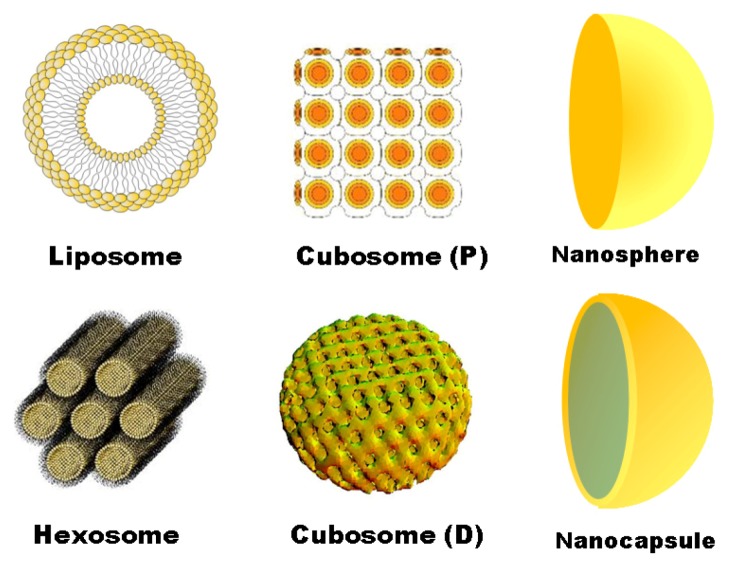
Lipid nanoparticulate carriers produced by soft nanotechnology. The channels allowing for protein nanoencapsulation are organized on an inner hexagonal lattice in the hexosome lipid particles, whereas the cubosome lipid particles can have different inner channel symmetries, such as primitive (P) cubic (cubosome of P-type) and diamond (D) cubic lattices (cubosome of D-type).

Vesicles, liposomes, cubosomes, spongosomes and hexosome particles appear to be lipid-based vehicles suitable for peptide and protein encapsulation [282,289]. Such particles are biocompatible, biologically inert and show little toxicity and antigenic reactions. They can be prepared by hydration of a lipid film, followed by energy supply, for instance, mechanical vortexing and sonication. Upon agitation, the hydrated lipid sheets detach and self-assemble either in bilayer membrane-type liquid crystalline NPs or in lipid-monolayer-based structures (Figure 5). The diameter of the lipid vehicles can vary between 20 nm and several hundred microns. The stable, *in vivo* release of active molecules and biodistribution of the NPs are determined by their size, surface charge and lipid membrane fluidity. Sterically stabilized (“stealth”) liquid crystalline lipid NPs can be obtained by PEGylation or by self-assembly with amphiphilic copolymers, forming stabilizing hydrophilic shells. This may help avoiding aggregation during storage or due to physico-chemical instability. Targeted-delivery strategies can be realized by attachment of specific monoclonal antibodies or ligands to the lipid NPs surfaces [292]. 

Vesicles and liposomes are lipid membrane-type particles composed of one or more phospholipid bilayers enclosing an aqueous volume (Figure 5). Lipophilic substances can be incorporated into the lipid bilayers, while hydrophilic compounds can be entrapped in the aqueous core. Thus, the aqueous compartments can be used for the encapsulation of neurotrophins. 

Cubosomes and spongosomes are membrane-types of NPs with non-lamellar organizations. The cubosomes are characterized by periodically ordered cubic lattice membrane structures (of diverse symmetries, such as double diamond (D), gyroid (G) or primitive (P) cubic types), whereas spongosomes involve a random 3D membrane organization. Cubosome NPs have been considered to be more stable lipidic particulate systems, as compared to liposomes. In cubosome structures, the cubic lipid membrane subdivides the space into intertwined networks of aqueous channels. The enhanced capacity of these non-lamellar, multicompartment-type lipid vehicles for biomolecular drug encapsulation results from the liquid crystalline bicontinuous membrane architecture of the NPs. Compared to liposomes, the specific surface area developed by the lipid bilayer in non-lamellar-type lipid nanocarriers is much greater, thus increasing the possibilities for incorporation of guest molecules in cubosomes and spongosomes. These structures offer the opportunity to entrap neurotrophins in the water channels of the carriers, as well as for controlled release, through a slow diffusion process, from the nanochannelled assemblies. Cubosomes have been efficient in enhancement of the therapeutic protein stability against enzymatic degradation. Toward parenteral administration, colloidal dispersions of cubosome particles have been stabilized by the inclusion of an amphiphilic copolymer, such as Pluronic F127 [269,273]. Investigations on the interaction of cubosomes with plasma components in rats have shown a prolonged circulation of the particles [280]. On the other side, cubosomes have provided a new method for entrapment of protein molecules in lipid nanochannelled networks, leading to a new class of particles named “proteocubosomes” [265]. The proteocubosome carriers appear to be built up by assemblies of porous droplets (“nanocubosomes”) with diameters between 30 and 60 nm. Large protein molecules have been suggested to be confined at the interfaces between the nanocubosomes, inside the multicompartment proteocubosome particles.

Cubosomes modified by odorranalectin (a novel non-immunogenic small peptide) have been prepared as nanovehicles with mucoadhesive properties providing enhanced drug delivery to the brain upon intranasal administration [259]. In this intranasal route for treatment of CNS disorders, Gly14-humanin has been loaded, as a model neuropeptide drug, in cubosomes and evaluated for its pharmacodynamics in Alzheimer’s disease (AD) in rats. Based on the enhanced therapeutic effects found with the peptide-loaded cubosomes, the results have suggested that functionalized cubosomes represent a novel effective and non-invasive system for neurotrophic peptide transport [259]. 

Towards application in neurosurgery, triglyceride matrices have been examined for their biocompatibility and controlled release of BDNF [120]. In addition, concepts for novel lipid carriers for biomedical and diagnostic applications have been proposed, taking into account the ability of omega-3 polyunsaturated fatty acids (e.g., EPA) for neurotrophin potentiation [219]. 

Hexosomes are lipid NPs of inverted-hexagonal-phase inner arrangement of aqueous channels (Figure 5). The quantity of nanoencapsulated molecules in such carriers can be controlled via the diameter of the inner aqueous channels. The high surface-to-volume ratio appears to be advantageous for interaction of these NPs with cells. They have found applications as nanodispersions for local delivery of peptides and recently have been employed for neurotrophic factor encapsulation [293]. Another class of nanostructures presenting very interesting properties for brain diagnostics and neuroprotection are the magnetoliposomes [294,295,296]. Magnetic liposomes and NPs usually constitute multifunctional nanocarriers and have shown promises in both diagnostics and therapy. Magnetic carriers can be manipulated by an external magnet and directed towards the desired targeted site (“magnetic targeting”). In addition, they can promote the nanocarrier/cell interaction and internalization (“magnetofection”). Therefore, next-generation nanotherapeutic strategies, involving multifunctional lipid NPs, could be envisaged to increase the therapeutic index of the neurotrophin drugs. Before defining the advantages and shortcomings of such new systems, future research work will be required to address the questions about the best delivery way, time of sustain release, challenges of delivery to the brain, regulation of BDNF release at individual synapses, *etc.*

## 4. Conclusion

Preclinical and clinical studies have shown the benefits of the neurotrophic proteins and their limitations related to the employed means of administration. Major problems in neurotrophin delivery have been related to the fact that the administered protein has not reached the degenerated and damaged neurons. Intravenous administration of neurotrophins has been inefficient, because of low permeability of the BBB to these protein molecules. Vectorization of neurotrophins can extend their circulation time and improve their bioavailability by avoiding rapid degradation and elimination from the brain. The neurotrophin half-life has been prolonged to 7–14 days using polymer carriers (microspheres, scaffolds) and even to 3–4 weeks by means of hydrogel delivery systems. The copolymer PLGA-PLL-PEG has enabled delivery of therapeutic BDNF quantities up to 60 days in *in vitro* studies. Blood-brain barrier penetration has been observed predominantly with receptor-mediated (Trojan horse) technologies and small-molecule non-peptide mimetics of BDNF. Peptides, mimicking the neurotrophic factor structure, have shown partial agonists actions on the TrkB receptor and have appeared to be a promising emerging strategy for treatment of neurodegenerative diseases. 

It has been suggested that local long-term delivery of BDNF is necessary for beneficial neuroregenerative response. Various biocompatible carriers have been proposed for nondestructive local neurotrophin delivery and for potential therapies of neurodegenerative disorders. The strategies for local neurotrophin administration have often been based on invasive surgery. Cellular delivery has raised immunogenicity problems. However, clinical trials with encapsulated cell biodelivery (ECB) implants for targeted delivery of NGF have given strong promise in restorative neurosurgery of patients with Alzheimer’s disease. Moreover, gene therapy has considerably advanced in recent years and has demonstrated the possibilities for reversal of neurological damages and for stopping of neurodegeneration. Transfers of the *BDNF* gene have been of therapeutic value for Huntington’s disease and Alzheimer’s disease. In perspective, non-viral gene carriers (lipoplexes, polyplexes) can be anticipated to be confirmed as advantageous over viral gene carriers, because they are non-oncogenic, non-immunogenic and easy to produce over large scales. Analysis shows that the nose-to-brain and skin-to-brain interfaces have not been sufficiently explored as non-invasive delivery strategies. This may be related to the fact that the involved mechanisms and pharmacokinetics have not been fully elucidated yet. 

The fast-developing area of nanoparticle-based medicine holds the promise for emergence of new therapies for the treatment of neurodegenerative and psychiatric disorders. The neurotrophin-synthetic-NP carrier pharmacokinetics and mechanisms for BBB penetration have been scarcely studied in detail in the available reports. To avoid side effects, nanoscale delivery systems favoring targeted delivery in specific brain areas and minimizing the biodistribution to the systemic circulations should be preferably developed. Among them, it seems worth pursuing the studies on multicompartment liquid crystalline lipid NPs, incorporating omega-3 polyunsaturated fatty acids, which have been effective in promoting neurite growth and inhibition of neuronal cell apoptosis. 
